# Prevalence of Chronic Kidney Diseases in Patients with Diabetes Mellitus in the Middle East: A Systematic Review and Meta-Analysis

**DOI:** 10.1155/2021/4572743

**Published:** 2021-08-28

**Authors:** Abdallah Y Naser, Hassan Alwafi, Basil Alotaibi, Emad Salawati, Mohammed Samannodi, Zahra Alsairafi, Abeer F. R. Alanazi, Mohammad S. Dairi

**Affiliations:** ^1^Department of Applied Pharmaceutical Sciences and Clinical Pharmacy, Faculty of Pharmacy, Al-Isra University, Amman, Jordan; ^2^Faculty of Medicine, Umm Al Qura University, Mecca, Saudi Arabia; ^3^College of Pharmacy, King Saud University, Riyadh, Saudi Arabia; ^4^Department of Family Medicine, Faculty of Medicine, King Abdulaziz University, Jeddah, Saudi Arabia; ^5^Faculty of Pharmacy, Pharmacy Practice Department, Kuwait University, Kuwait City, Kuwait; ^6^Department of Pharmaceutical and Biological Sciences, UCL School of Pharmacy, London, UK

## Abstract

**Aims:**

The prevalence of CKD in patients with diabetes mellitus in the Middle East region is unknown. Therefore, we aimed to understand the pooled prevalence of CKD in patients with diabetes mellitus in the Middle East region.

**Methods:**

PubMed, Embase, and Cochrane databases were searched for relevant studies up to October 2020. The search strategy was conducted using both keywords and MeSH terms. Randomised controlled trials (RCTs) and observational studies that included patients from all age groups and any study design that reported on the prevalence of CKD in patients with diabetes mellitus were included. The pooled estimate for the prevalence of CKD in patients with diabetes was calculated using random-effect models with 95% confidence intervals (CIs).

**Results:**

A total of 489 citations were identified, of which only nine studies matched our inclusion criteria and were included in the meta-analysis. All of the studies used an observational study design covering a total of 59,395 patients with type 2 diabetes mellitus. The pooled estimate of the prevalence of CKD in patients with diabetes mellitus was 28.96% (95% CI: 19.80–38.11).

**Conclusions:**

A high prevalence of CKD in patients with diabetes mellitus in the Middle East region was found. Further epidemiological studies are warranted in this area to have a better estimate of the prevalence of CKD among DM in the Middle East region.

## 1. Introduction

Diabetes mellitus (DM) is a multifactorial disease, where the body becomes unable to utilise or produce a sufficient amount of insulin to match the body's needs [1]. Medical guidelines promote optimal glycaemic control to prevent the progress of the disease and the development of microvascular and macrovascular complications among patients with DM [2–6]. One of the main macrovascular compilations of DM is nephropathy or chronic kidney disease (CKD) [7].

DM is considered an initiation factor that directly results in kidney damage. In addition, it is one of the main progression factors that is associated with a further decline in the kidney function [8]. One of the main challenges of diagnosing CKD is that it is asymptomatic among the patients with stages 1 and 2, and metabolic derangements start to be seen at stages 3, 4, and 5 [9].

CKD is considered one of the leading causes of morbidity and mortality worldwide [10]. It is commonly diagnosed using an albumin-to-creatinine ratio (ACR), by imaging the kidneys, and by kidney function tests [11, 12]. CKD has a high global prevalence of around 11% to 13%, and diabetes prevalence increased dramatically in the Eastern Mediterranean countries in the past 25 years [13].

CKD is associated with an increased risk of hospitalisation and a decrease in patients' quality of life [14–17]. The morbidity and mortality risk is even higher in patients with DM as the disease itself participates in the progress of other comorbidities due to its effect on body organs [17]. Previous systematic reviews and meta-analyses have focused on the prevalence of CKD in the general population or other regions [13, 18]. However, no previous meta-analysis has investigated the prevalence of CKD in patients with DM in the Middle East region. Therefore, a better understanding of the prevalence of CKD in patients with DM in the Middle East is needed to establish baseline data on the prevalence of CKD in patients with DM.

## 2. Methods

The Preferred Reporting Items for Systematic Review and Meta-Analysis (PRISMA) guidelines were used as a guide to conduct the search process in order to guarantee the careful planning of the search and the consistency of search implementation [19]. In addition, this study was reported following the Meta-analysis of Observational Studies in Epidemiology (MOOSE) guidelines. The protocol of this systematic review and meta-analysis was registered in the PROSPERO database (PROSPERO CRD42019125162) [20].

### 2.1. Search Strategy and Study Selection

We conducted an electronic search in PubMed, Embase, and the Cochrane library to identify relevant published studies on the prevalence of CKD in patients with DM in the Middle East region up to January 16, 2019, without any language restrictions. Additionally, the reference list of the included studies was also scanned to identify further eligible studies. To answer the review question, the keywords of the search strategy focused on the PICO framework [21]. Based on this, the following keywords were used in the search process: “Prevalence OR Incidence” AND “Diabetes mellitus” AND “Chronic Kidney Disease” AND “Middle East,” taking into consideration different synonyms for each keyword and suffix variation, which is covered through the use of “truncation characters,” and combining the search results with the search results of the “MeSH term, subject heading, thesaurus, or MeSH tree.” EndNote X7 software was used to import and manage the output of the database searches.

The inclusion criteria comprised studies that reported the percentage of diabetes mellitus patients with CKD, which is defined as kidney function or architecture abnormalities, a low level of glomerular filtration rate, or the existence of kidney damage markers such as albuminuria/proteinuria, urine sediment or electrolyte abnormalities, histological or structural anomalies by imaging studies, or kidney transplant history [22]. Moreover, CKD is classified based on albumin excretion rate (AER) or albumin-to-creatinine ratio (ACR) [22] and is diagnosed by calculating the GFR and the presence of proteinuria by measuring the urinary albumin/creatinine ratio [23]. Randomised controlled trial (RCT) or nonrandomized studies including cross-sectional, cohort, and case-cohort studies that reported prevalence data or provided sufficient data to calculate the prevalence of CKD in patients with DM from countries in the Middle East were included. Studies were excluded if they were on gestational diabetes, animal studies, or review articles. Two reviewers (HA and BA) screened the titles and abstracts of all identified studies independently, with any disagreement resolved by a third reviewer (AN). Studies not related to the topic and deemed irrelevant were excluded. Eligibility for the studies was judged based on the inclusion and exclusion criteria. Full-text studies were obtained for the assessment process.

### 2.2. Data Extraction and Quality Assessment

Two reviewers (HA and BJ) independently extracted data from the included studies using the data extraction form. Data extraction was checked by a third reviewer (SH). The extracted information included (1) authors' details (names and year of publication); (2) study characteristics (country, study design, setting, and size of the study population); (3) participants' characteristics (age, gender, and type of diabetes); (4) CKD characteristics (CKD diagnostic criteria and the number of participants diagnosed with CKD).

Using the Modified Newcastle–Ottawa Scale for Observational Studies [24], two reviewers (BJ and AN) independently assessed the included studies for their methodological quality. The quality assessment involved checking for five criteria: representativeness of the population, sample size, statistical analysis, ascertainment of outcomes, and comparability. A study can obtain a maximum of five points on this scale. Studies with ≥3 points were considered as high-quality studies, while studies with <3 points were considered as low-quality studies [24].

### 2.3. Data Synthesis

The prevalence rate of CKD in patients with DM with a 95% confidence interval was calculated (number of cases/sample size) based on the information on crude numerators and denominators provided in each study. All analyses were done using Stata v15. The pooled estimate for the prevalence of CKD in patients with diabetes was calculated using random-effect models with 95% confidence intervals (CIs). The function metan was used for the analysis of prevalence. Homogeneity was assessed using the Cochrane *Q*-test, with *p* < 0.10 [25]. The degree of heterogeneity was estimated by *I*^2^. *I*^2^ value <25% indicated low heterogeneity, 25–75% moderate, and >75% high heterogeneity [25]. Publication bias was assessed using funnel plots.

## 3. Results

**Figure 1:**
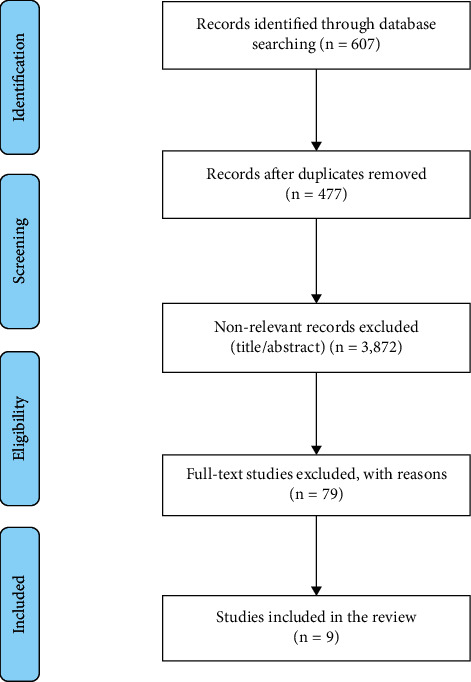
PRISMA flow diagram showing the study selection process.

A total of 607 studies were identified from the database search. After removing duplicates (*n* = 130), the remaining 477 studies were scanned for the titles and abstracts, of which 79 were selected for full-text screening. Nine studies met the inclusion criteria. The interrater agreement for inclusion was excellent (100%). The selection process of the studies was summarised using the PRISMA flow diagram [26] (Figure 1).

The details of the included studies are summarised in Table 1. The included studies were published between 2009 and 2018. Three studies were conducted in Saudi Arabia [27, 33, 34], while two studies were conducted in Oman [28, 29], and the remaining studies were conducted across different regions of the Middle East. Data from 59,395 participants were included, with the mean age ranging between 50.8 and 66.9 years. All studies investigated the prevalence of CKD among patients with T2DM, while one study was conducted on patients with T1DM and T2DM.

### 3.1. Prevalence of CKD

**Figure 2:**
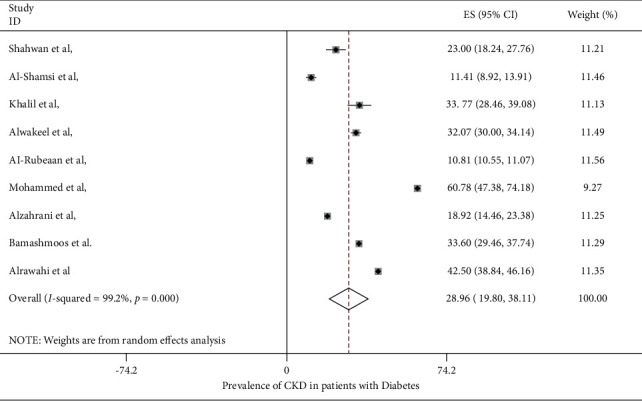
Forest plot showing the prevalence of CKD in T2DM patients.

**Figure 3:**
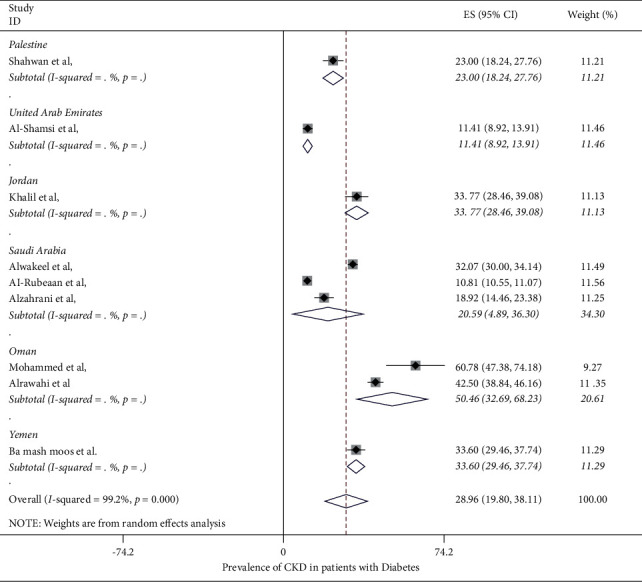
Forest plot showing the prevalence of CKD in T2DM patients stratified by location.

**Figure 4:**
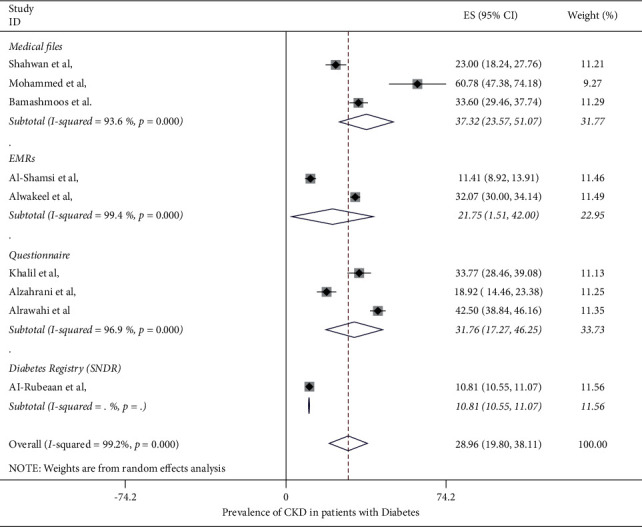
Forest plot showing the prevalence of CKD in T2DM patients stratified by the data source.

The prevalence of CKD in patients with diabetes mellitus in the Middle East region ranged from 10.8% (95% CI: 10.6%–11.1%) to 60.78% (95% CI: 47.38%–47.18%) in the included studies, with the overall pooled average of prevalence of CKD in patients with DM being 28.96% (95% CI: 19.80%–38.11%) (Figure 2). The pooled average of the prevalence of CKD in patients with DM stratified by location was 20.59% (95% CI: 4.98%–36.30%) in Saudi Arabia, while it was higher in Oman at 50.46% (95% CI: 32.69%–68.23%) (Figure 3). When stratifying the studies by the data source, the pooled average of the prevalence of CKD in patients with DM ranged 37.32% (95% CI: 32.57%–51.07%) in studies using medical files and 31.76% (95% CI: 17.27%–46.25%) in studies using self-reporting questionnaires, while it was lower in studies using EMRs (21.75%) (95% CI: 1.51%–42.0%) (Figure 4). A study conducted by Stroup et al. [20] investigated the prevalence rate of CKD by gender and found that males were at a higher risk of CKD compared to females, with a prevalence rate of 11.6% (95%CI: 11.2%–12.0%) and 9.8 (95%CI: 9.4%–10.2%).

### 3.2. Quality of Studies Included

The included studies were found to have high quality according to the score attainment on a modified version of the Newcastle–Ottawa scale (Table 2).

### 3.3. Publication Bias

**Figure 5:**
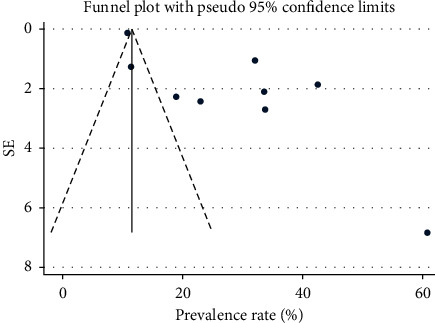
Funnel plot showing evidence of publication bias.

The evidence from the funnel plot shows that there was a source of publication bias in the present study. The findings resembled asymmetrical funnel plots (details are presented in Figure 5).

## 4. Discussion

Our systematic review and meta-analysis investigated the prevalence of CKD in patients with diabetes mellitus in the Middle East. The result of this study demonstrates that the prevalence is high in patients with diabetes. Previous systematic review and meta-analyses investigating the prevalence of CKD in patients with DM in the Middle East are lacking. However, previous research has investigated the prevalence of CKD in patients with DM in the general population or in other countries. A large population-based study in China reported around 35.5% of CKD prevalence in patients with diabetes [35]. Similarly, a recent systematic review from the African continent reported a pooled prevalence of CKD of around 24.7% (95% CI: 23.6%–25.7%) in patients with diabetes and 10.1% (95% CI: 9.8%–10.5%) in the general population [36].

CKD is a common complication of DM, especially when patients have uncontrolled blood sugar levels. Patients with diabetes have a higher risk of developing CKD. Type 2 diabetes mellitus and a longer duration of diabetes were considered as independent risk factors of CKD with an adjusted odd ratio (OR) of 3.75 (95% CI: 1.50–9.33) and OR of 3.38 (95% CI: 1.39–8.19) [37]. Risk stratification according to sex reveals that CKD associated with DM was reported to be 3.34 (95% CI: 2.27–4.93) in women and 2.84 (95% CI: 1.73–4.68) in men [38].

Our results showed that there was a considerable variation in the data source. Studies that were based on a self-reported or a medical file data source had higher prevalence rates of CKD compared to studies that were based on health record databases. This could likely be because of the way individual questions are designed on self-reported studies, because of barriers in communication, and because self-reported studies may lack an accurate diagnosis. This is also influenced by the precision of individual reviews, which can be poor [39]. On the contrary, health record databases are more likely to report more accurate diagnoses that require either emergency visits or hospital admissions, in which the events are confirmed by healthcare professionals.

DM is associated with different comorbidities, and patients face a higher risk of developing CKD when multiple comorbidities coexist. Cardiovascular disease affects 32.2% of people with T2DM globally [40]. CVD is also associated with CKD [41, 42]. In addition, patients with diabetes are likely to be prescribed more than one type of medication; therefore, patients with diabetes are at a higher risk of CKD due to the effect of polypharmacy [43–45].

Although Middle Eastern countries are considered as developing countries, some of the countries in the region have good healthcare systems with easy access for citizens. However, the Middle East also has a unique cultural and behavioural element that may be linked to several noncommunicable diseases, including DM and CKD. It is, therefore, imperative to say that the high number of CKD in patients with DM in the Middle East could be due to several factors and not only due to the independent risk of CKD in patients with diabetes.

Diabetes is a multisystem disease, which is associated with multiple complications [24]. Similarly, CKD is associated with poor quality of life and significant morbidity and mortality [46–48]. Therefore, patients that have both diseases could have a higher risk of morbidity and mortality [49].

Health organisations and high-level policymakers in Middle Eastern countries should be aware of these high numbers, and more public awareness should be raised to minimise the risk of having both diseases together. However, it is important to mention that few studies have focused on both conditions in the Middle East, and more research is needed to fill this knowledge gap.

### 4.1. Strengths and Limitations

This study is the first meta-analysis that investigates the prevalence of CKD in patients with diabetes in the Middle East. In addition, the strengths of the present meta-analysis include a protocol-oriented approach, pooled analysis based on high-quality studies, and an extensive literature search. However, this study also has some limitations. One limitation of this research is that it includes only a limited number of studies; however, we tried to devise a broad search strategy by searching in three different databases. High heterogeneity was observed in this meta-analysis, which was not explained by any of the study-level covariates considered in the subgroup analysis. Therefore, the high heterogeneity is likely to be due to study characteristics that were not measured or reported in the original citations [50]. In addition, it is important to highlight that high statistical heterogeneity is more frequent in meta-analyses of prevalence compared to meta-analyses of binary outcomes [51]. This could be for multiple reasons other than clinical variations, such as variations in the methodologies, randomisation, the sample size of the included studies, the source of the data, the geographical locations, and the methodology of the studies [51].

## 5. Conclusion

A high prevalence of CKD was found among DM patients in the Middle East region. Very few studies have investigated the prevalence of CKD among patients with diabetes in the Middle East region. Further studies are warranted to have a better prevalence estimate. The government should frame policies for targeted screening of kidney function among high-risk group patients to prevent this.

## Figures and Tables

**Table 1:** Study design characteristics of included studies.

Author	Year of publication	Design	Data source	Type of diabetes	CKD diagnostic criteria	Country	Population	Mean age (SD)	Gender	Number of patients having CKD
Moher et al. [19]	2009	Retrospective cross-sectional	Electronic medical records	T2DM	Proteinuria, SCr, and eGFR	Saudi Arabia	1,952	66.9 ± 11.4	47% (294) were males and 53% (332) were females	626

Stroup et al. [20]	2014	Retrospective cross-sectional	Diabetes registry (SNDR)	T2DM	Proteinuria, SCr, eGFR, and albumin excretion	Saudi Arabia	54,670	59.9 ± 12.7	59.5% (3518) were males and 40.5% (2394) were females	5,912

Alzahrani et al. [27]	2019	Cross-sectional	Questionnaire	T2DM	eGFR, ACR	Saudi Arabia	296	60.29 ± 10.593	23.4% (26) were males and 16.2% (30) were females	296

Alrawahi et al. [28]	2012	Cross-sectional	Questionnaire	T2DM	Albuminuria/creatinine	Oman	699		51.6% were males	699

Mohammed et al. [29]	2018	Cross-sectional	Medical files	T2DM	Proteinuria, SCr, eGFR, and albumin excretion	Oman	51	50.8	54.9% males and females 45.1%	51

Schardt et al. [21]	2013	Prospective	Hospital	T2DM	ADA criterion	Yemen	500	56.3 ± 32		168

Jamal Shahwan et al. [30]	2019	Cross-sectional	Medical files	T2DM	Creatinine, glomerular filtration rate (GFR), creatinine urine, albumin/creatinine ratio	Palestine	300	57 ± 8.5	54% (162) were males and 46% (138) were females	300

Al-Shamsi et al. [31]	2019	Retrospective cohort study	Electronic medical records	T2DM	SCr and eGFR	United Arab Emirates	622	52.38 ± 14.48	50% were males (312)	622

Khalil et al. [32]	2017	Cross-sectional	Questionnaire	T2DM	eGFR	Jordan	305	55.0 ± 12.5	64% were females (347)	305

ADA: American Diabetes Association; T2DM: type 2 diabetes mellitus.

**Table 2:** Quality assessment of included studies.

Author (ref), year of the study	Representativeness of the sample	Sample size	Comparability (non-CKD)	Outcome (ascertainment of CKD)	Statistics	Total	Quality
Moher et al., 2009 [19]	Yes	Yes	No	No	Yes	3	High
Stroup et al., 2014 [20]	Yes	Yes	Yes	Yes	Yes	4	High
Schardt et al., 2013 [21]	Yes	Yes	No	Yes	Yes	4	High
Alzahrani et al. [27]	Yes	Yes	Yes	Yes	Yes	4	High
Alrawahi et al. [28]	Yes	Yes	Yes	Yes	Yes	4	High
Mohammed et al. [29]	Yes	Yes	Yes	Yes	Yes	4	High
Schardt et al. [21]	Yes	Yes	Yes	Yes	Yes	4	High
Jamal Shahwan et al. [30]	Yes	Yes	Yes	Yes	Yes	4	High
Al-Shamsi et al. [31]	Yes	Yes	Yes	Yes	Yes	4	High
Khalil et al. [32]	Yes	Yes	Yes	Yes	Yes	4	High

## Data Availability

Data sharing is not applicable to this article as the data are publicly available.

## Additional Points

*What Is Already Known about This Subject*? Diabetes is a highly prevalent disease in the Middle East and worldwide. Patients with diabetes have an increased risk of developing chronic kidney disease (CKD). Limited studies investigate the prevalence of CKD in patients with diabetes in the Middle East. *What Does This Article Add*? High prevalence of CKD was found among patients with diabetes in the Middle East region. This study found limited research on the prevalence of CKD in patients with diabetes in the Middle East. The prevalence of CKD in patients with diabetes in the Middle East is considerably high. CKD is more common among male diabetic patients. The prevalence of CKD in patients with diabetes is higher in self-reporting studies compared to electronic health record studies.

## References

[1] National Institute of Diabetes and Digestive and Kidney Diseases (NIDDK) (2014). *Causes of Diabetes*.

[2] Hanssen K. F. (1997). Blood glucose control and microvascular and macrovascular complications in diabetes. *Diabetes*.

[3] Avignon A., Radauceanu A., Monnier L. (1997). Nonfasting plasma glucose is a better marker of diabetic control than fasting plasma glucose in Type 2 diabetes. *Diabetes Care*.

[4] Polonsky K. S., Given B. D., Hirsch L. J. (1988). Abnormal patterns of insulin secretion in non-insulin-dependent diabetes mellitus. *New England Journal of Medicine*.

[5] Wong M. G., Perkovic V., Chalmers J. (2016). Long-term benefits of intensive glucose control for preventing end-stage kidney disease: advance-on. *Diabetes Care*.

[6] LeRoith D., Rayfield E. J. (2007). The benefits of tight glycemic control in type 2 diabetes mellitus. *Clinical Cornerstone*.

[7] Yokoyama H., Araki S.-I., Kawai K. (2018). Declining trends of diabetic nephropathy, retinopathy and neuropathy with improving diabetes care indicators in Japanese patients with type 2 and type 1 diabetes (JDDM 46). *BMJ Open Diabetes Research & Care*.

[8] Kazancioglu R. (2013). Risk factors for chronic kidney disease: an update. *Kidney International Supplements*.

[9] Levey A. S., Coresh J. (2012). Chronic kidney disease. *Lancet (London, England)*.

[10] Neuen B. L., Chadban S. J., Demaio A. R., Johnson D. W., Perkovic V. (2017). Chronic kidney disease and the global NCDs agenda. *BMJ Global Health*.

[11] National Institue for Health and Care Excellence (2008). *Chronic Kidney Disease: Early Identification and Management of Chronic Kidney Disease in Adults in Primary and Secondary Care*.

[12] National Kidney Foundation (2019). *About Chronic Kidney Disease*.

[13] Hill N. R., Fatoba S. T., Oke J. L. (2016). Global prevalence of chronic kidney disease-a systematic review and meta-analysis. *PLoS One*.

[14] Gansevoort R. T., Correa-Rotter R., Hemmelgarn B. R. (2013). Chronic kidney disease and cardiovascular risk: epidemiology, mechanisms, and prevention. *The Lancet*.

[15] Go A. S., Chertow G. M., Fan D., McCulloch C. E., Hsu C.-Y. (2004). Chronic kidney disease and the risks of death, cardiovascular events, and hospitalization. *New England Journal of Medicine*.

[16] Perlman R. L., Finkelstein F. O., Liu L. (2005). Quality of life in chronic kidney disease (CKD): a cross-sectional analysis in the Renal Research Institute-CKD study. *American Journal of Kidney Diseases*.

[17] Chin H. J., Song Y. R., Lee J. J. (2008). Moderately decreased renal function negatively affects the health-related quality of life among the elderly Korean population: a population-based study. *Nephrology Dialysis Transplantation*.

[18] Anothaisintawee T., Rattanasiri S., Ingsathit A., Attia J., Thakkinstian A. (2009). Prevalence of chronic kidney disease: a systematic review and meta-analysis. *Clinical Nephrology*.

[19] Moher D., Liberati A., Tetzlaff J., Altman D. G. (2009). Preferred reporting items for systematic reviews and meta-analyses: the PRISMA statement. *PLoS Medicine*.

[20] Stroup D. F., Berlin J. A., Morton S. C. (2000). Meta-analysis of observational studies in epidemiology a proposal for reporting. *Journal of the American Medical Association*.

[21] Schardt C., Adams M. B., Owens T., Keitz S., Fontelo P. (2007). Utilization of the PICO framework to improve searching PubMed for clinical questions. *BMC Medical Informatics and Decision Making*.

[22] Stevens P. E., Levin A. (2013). Evaluation and management of chronic kidney disease: synopsis of the kidney disease: improving global outcomes 2012 clinical practice guideline. *Annals of Internal Medicine*.

[23] Vassalotti J. A., Stevens L. A., Levey A. S. (2007). Testing for chronic kidney disease: a position statement from the National Kidney Foundation. *American Journal of Kidney Diseases*.

[24] Hussain S., Habib A., Singh A., Akhtar M., Najmi A. K. (2018). Prevalence of depression among type 2 diabetes mellitus patients in India: a meta-analysis. *Psychiatry Research*.

[25] Higgins J. P. T., Thompson S. G., Deeks J. J., Altman D. G. (2003). Measuring inconsistency in meta-analyses. *BMJ*.

[26] Moher D., Liberati A., Tetzlaff J., Altman D. G. (2009). Preferred reporting items for systematic reviews and meta-analyses: the PRISMA statement. *PLoS Medicine*.

[27] Alzahrani B., Mohammed Alturkistani A., Alzidani T., Alturkistani A., Abozaid H. (2019). Prevalence and risk factors for diabetic nephropathy in type 2 diabetic patients, Taif City, Saudi Arabia. *International Journal of Medicine in Developing Countries*.

[28] Alrawahi A. H., Rizvi S. G. A., Al-Riyami D., Al-Anqoodi Z. (2012). Prevalence and risk factors of diabetic nephropathy in omani type 2 diabetics in Al-dakhiliyah region. *Oman Medical Journal*.

[29] Mohammed E., Atris A., Al Salmi I., Al-Menawi L., Shaheen F., Hannawi S. (2018). Clinical and laboratory findings of patients with diabetes undergoing kidney biopsy. *Saudi Journal of Kidney Diseases and Transplantation: An Official Publication of the Saudi Center for Organ Transplantation, Saudi Arabia*.

[30] Jamal Shahwan M., Hassan N. A. G., Shaheen R. A. (2019). Assessment of kidney function and associated risk factors among type 2 diabetic patients. *Diabetes & Metabolic Syndrome: Clinical Research & Reviews*.

[31] Al-Shamsi S., Oulhaj A., Regmi D., Govender R. D. (2019). Use of estimated glomerular filtration rate to predict incident chronic kidney disease in patients at risk of cardiovascular disease: a retrospective study. *BMC Nephrology*.

[32] Khalil A. A., Abed M. A., Ahmad M., Mansour A. H. (2018). Under-diagnosed chronic kidney disease in Jordanian adults: prevalence and correlates. *Journal of Renal Care*.

[33] Alwakeel J. S., Al-Suwaida A., Isnani A. C., Al-Harbi A., Alam A. (2009). Concomitant macro and microvascular complications in diabetic nephropathy. *Saudi Journal of Kidney Diseases and Transplantation: An Official Publication of the Saudi Center for Organ Transplantation, Saudi Arabia*.

[34] Al-Rubeaan K., Youssef A. M., Subhani S. N. (2014). Diabetic nephropathy and its risk factors in a society with a type 2 diabetes epidemic: a Saudi National Diabetes Registry-based study. *PLoS One*.

[35] Duan J., Wang C., Liu D. (2019). Prevalence and risk factors of chronic kidney disease and diabetic kidney disease in Chinese rural residents: a cross-sectional survey. *Scientific Reports*.

[36] Abd ElHafeez S., Bolignano D., D’Arrigo G., Dounousi E., Tripepi G., Zoccali C. (2018). Prevalence and burden of chronic kidney disease among the general population and high-risk groups in Africa: a systematic review. *BMJ Open*.

[37] Damtie S., Biadgo B., Baynes H. W. (2018). Chronic kidney disease and associated risk factors assessment among diabetes mellitus patients at a tertiary hospital, northwest Ethiopia. *Ethiopian journal of health sciences*.

[38] Shen Y., Cai R., Sun J. (2017). Diabetes mellitus as a risk factor for incident chronic kidney disease and end-stage renal disease in women compared with men: a systematic review and meta-analysis. *Endocrine*.

[39] Choi B. C. K., Pak A. W. P. (2005). A catalog of biases in questionnaires. *Preventing Chronic Disease*.

[40] Einarson T. R., Acs A., Ludwig C., Panton U. H. (2018). Prevalence of cardiovascular disease in type 2 diabetes: a systematic literature review of scientific evidence from across the world in 2007-2017. *Cardiovascular Diabetology*.

[41] Matsushita K., van der Velde M., Astor B. C. (2010). Association of estimated glomerular filtration rate and albuminuria with all-cause and cardiovascular mortality in general population cohorts: a collaborative meta-analysis. *The Lancet*.

[42] Hussain S., Siddiqui A. N., Baxi H., Habib A., Hussain M. S., Najmi A. K. (2019). Warfarin use increases bleeding risk in hemodialysis patients with atrial fibrillation: a meta‐analysis of cohort studies. *Journal of Gastroenterology and Hepatology*.

[43] Schmidt I. M., Hübner S., Nadal J. (2019). Patterns of medication use and the burden of polypharmacy in patients with chronic kidney disease: the german chronic kidney disease study. *Clinical Kidney Journal*.

[44] Alwafi H., Wei L., Naser A. Y. (2020). Trends in oral anticoagulant prescribing in individuals with type 2 diabetes mellitus: a population-based study in the UK. *BMJ Open*.

[45] Alwafi H., Wong I. C. K., Banerjee A. (2020). Epidemiology and treatment of atrial fibrillation in patients with type 2 diabetes in the UK, 2001-2016. *Scientific Reports*.

[46] Hussain S., Habib A., Najmi A. K. (2019). Anemia prevalence and its impact on health‐related quality of life in Indian diabetic kidney disease patients: evidence from a cross‐sectional study. *Journal of Evidence-Based Medicine*.

[47] Port F. K., Fenton S. S. A., Mazzuchi N. (2000). ESRD throughout the world: morbidity, mortality, and quality of life. *Kidney International*.

[48] Hussain S., Habib A., Najmi A. K. (2019). Limited knowledge of chronic kidney disease among type 2 diabetes mellitus patients in India. *International Journal of Environmental Research and Public Health*.

[49] Hussain S., Habib A., Najmi A. K. (2018). A16804 pulmonary hypertension increases the risk of all-cause mortality in dialysis patients: evidence-based meta-analysis using real world data. *Journal of Hypertension*.

[50] Barendregt J. J., Doi S. A., Lee Y. Y., Norman R. E., Vos T. (2013). Meta-analysis of prevalence. *Journal of Epidemiology and Community Health*.

[51] Alba A. C., Alexander P. E., Chang J., MacIsaac J., DeFry S., Guyatt G. H. (2016). High statistical heterogeneity is more frequent in meta-analysis of continuous than binary outcomes. *Journal of Clinical Epidemiology*.

